# Ultrasound Biomicroscopy for Longitudinal Studies of Carotid Plaque Development in Mice: Validation with Histological Endpoints

**DOI:** 10.1371/journal.pone.0029944

**Published:** 2012-01-05

**Authors:** Erin Y. Harmon, Van Fronhofer, Rebecca S. Keller, Paul J. Feustel, M. Julia Brosnan, Jan H. von der Thüsen, Daniel J. Loegering, Michelle R. Lennartz

**Affiliations:** 1 Center for Cell Biology and Cancer Research, Albany Medical College, Albany, New York, United States of America; 2 Center for Cardiovascular Sciences, Albany Medical College, Albany, New York, United States of America; 3 Center for Neuropharmacology and Neuroscience, Ordway Research Institute, Albany, New York, United States of America; 4 Center for Metabolic Disease, Ordway Research Institute, Albany, New York, United States of America; 5 Department of Pathology, Academic Medical Center, Amsterdam, The Netherlands; Heart Center Munich, Germany

## Abstract

Atherosclerosis is responsible for the death of thousands of Americans each year. The carotid constriction model of plaque development has recently been presented as a model for unstable plaque formation in mice. In this study we 1) validate ultrasound biomicroscopy (UBM) for the determination of carotid plaque size, percent stenosis, and plaque development in live animals, 2) determine the sensitivity of UBM in detecting changes in blood flow induced by carotid constriction and 3) test whether plaque formation can be predicted from blood flow parameters measured by UBM. Carotid plaques were induced by surgical constriction in Apo E−/− mice. Arteries were imaged bi-weekly by UBM, at which time PW-Doppler measurements of proximal blood flow, as well as plaque length and percent stenosis were determined. Histology was performed 9 weeks post surgery. When compared to whole mount post-mortem measurements, UBM accurately reported carotid plaque length. Percent stenosis, based on transverse B-mode UBM measurements, correlated well with that calculated from histological sections. PW-Doppler revealed that constriction reduced maximum systolic velocity (v_max_) and duration of the systolic velocity peak (t_s_/t_t_). Pre-plaque (2 week post-surgery) PW-Doppler parameters (v_max_ and t_s_/t_t_) were correlated with plaque length at 9 weeks, and were predictive of plaque formation. Correlation of initiating PW-Doppler parameters (v_max_ and t_s_/t_t_) with resulting plaque length established the degree of flow disturbance required for subsequent plaque development and demonstrated its power for predicting plaque development.

## Introduction

Each day, atherosclerosis takes the lives of more than 2,200 Americans, with stroke accounting for ∼5% of those deaths [1]. Although major advances in our understanding of atherosclerosis have come from Apo E−/− and LDLR−/− atherosclerosis-prone mice, the majority of studies focus on plaques in aortic regions of the vasculature (ie. descending aorta and aortic root) with very limited data available on carotid plaques [2], the rupture of which lead to stroke. Indeed, carotid plaques progress slowly in mice, making the study of their development difficult. Two groups have reported the rapid induction of carotid plaques in mice by carotid constriction [3], [4]. Implantation of constrictive devices mimics the low shear stress that, in humans, contributes to carotid atherosclerosis [5]. Two geometries of constriction have been used: a constrictive cuff [3] and a conical cast [4], [6]. The plaques induced by carotid constriction are complex (i.e., contain macrophages, smooth muscle cells, and a lipid-rich core) and have histological features (e.g., thin cap, necrotic core, reduced collagen, and intra-plaque hemorrhage) similar to those of vulnerable human carotid plaques. Thus, carotid constriction induces a carotid atherosclerosis in mice with many of the features of vulnerable human plaques [3], [4].

Until recently, developmental studies have suffered from the necessity to sacrifice animals at varying ages to gather information on plaque size and stenosis. This situation changed with the advent of small animal, high-resolution ultrasound biomicroscopy (UBM). UBM is non-invasive, permitting longitudinal studies on plaque initiation, growth, and regression in the same animal that has been used to visualize spontaneously formed plaques in the brachiocephalic [7], [8] thoracic arteries [9], and the ascending aorta [10]. However, few studies have applied UBM to carotid plaques [11], [12] and fewer yet have exploited its non-invasive property to follow plaque development with time [13].

In the present study, we have employed UBM for *in vivo* determination of carotid plaque length and percent stenosis, and have documented plaque progression over time in ApoE−/− mice fitted with either a constrictive cast or cuff. Additionally, using PW-Doppler, we have identified two velocity parameters that accurately predict plaque formation. Collectively, these results validate UBM for both predicting carotid plaque formation and for the longitudinal study of its development.

## Methods

### Animals

**Figure 1:**
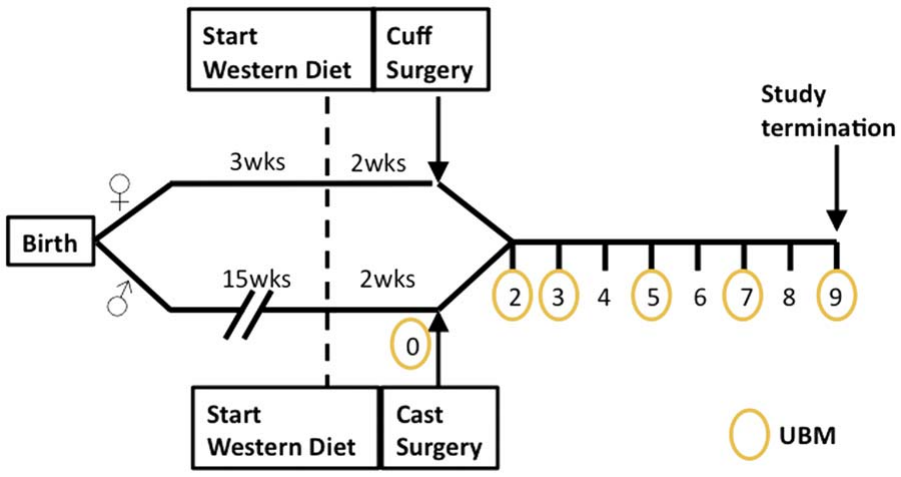
Study time-line.

All animal procedures were approved by the Albany Medical Center Institutional Animal Care and Use Committee and carried out in compliance with NIH regulations. ApoE−/− mice on the C57BL/6 background were purchased from The Jackson Laboratory (Bar Harbor, ME) (stock#002052) and bred in-house. Animals receiving constrictive cuffs were female and entered the study at 3 weeks of age (n = 13); Of these 13, 3 were removed from the study at 2 weeks (surgical occlusion rather than constriction), 3 were removed from the study due to complications resulting in no flow prior to the 9 week time-point, leaving a total of 7. Mice receiving conical casts were male and entered the study at 15 weeks (n = 14). Of these 14, 3 were removed from the study because of no flow resulting from surgical complications. One was removed due to complications resulting in no flow prior to the 9 week time-point (total casted animals = 10). At 3 or 15 weeks, animals were started on a high fat Western-type diet (0.25% cholesterol, 15% cocoa butter, 40% sucrose, Harlan Teklad, Madison, WI), available *ad libitum*. Cuffs and casts were placed at 5 or 17 weeks, respectively. See Fig. 1 for time-line.

### Cuff and cast devices

Medical grade platinum cured silicone tubing (0.305×0.635 mm, Degania Silicone, Inc, Cumberland, RI) was cut into 2 mm lengths and a longitudinal incision made to generate a cuff. Conical casts (cone: 0.2×0.1 mm, 1.5 mm in length) were purchased from Promodling BV (The Hague, Netherlands). Cuffs with an inner diameter of 0.3 mm and casts with a minimum inner diameter of 0.1 mm were chosen as these sizes were estimated to constrict the artery ∼70% [3], [4], which corresponds to the percent stenosis at which endarterectomies are recommended in humans. Cuffs and casts were sterilized under UV light (2 h) and stored in 70% ethanol. Surgeries were performed within 24 h of device sterilization.

### Device implantation

Cuffs and casts (hereafter referred to as “devices”) were placed on the right common carotid artery (RCCA) proximal to the bifurcation, according to published procedures [3], [4]. Briefly, the common carotid artery was exposed and separated from the vagus nerve. The constrictive device was placed around the artery where the middle thyroid vein crosses the RCCA; casts were tied with a single suture, cuffs with two. Animals were housed individually post-surgery.

### Ultrasound Biomicroscopy

All animals were subjected to pre-surgery ultrasound the week of device implantation. Following a two-week healing period, animals were imaged bi-weekly for the duration of the study (Fig. 1). Ultrasound was performed using the Vevo 770 high resolution imaging system (VisualSonics) 40 MHz transducer with the animals maintained under anesthesia (2% isoflurane inhalation). Hair was removed from the chest using a chemical depilatory (Veet, Reckitt Benckiser North America, Inc, Persippany, NJ). Aquasonic 100® ultrasound transmission gel (Parker Lab) was applied to the chest. Plaque length was measured using B-mode sagittal views. Percent stenosis was calculated from transverse images using the following equation:
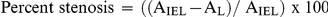

[14] where A_IEL_ is the area circumscribed by the inner elastic lamina and A_L_ is the luminal area. The device was used as a landmark to estimate placement within artery. The most stenotic region of the artery by UBM was correlated with the most stenotic region of histological preparations. Usually this region was immediately proximal to the device. The proximal flow paramaters (t_S_,t_T_, v_max_) were calculated using PW-Doppler measurements taken approximately 0.5 mm proximal to device margin. Beam-to-flow angle was minimized (<60°) for PW-Doppler measurements.

### Tissue harvesting

At 9 weeks post-surgery, animals were anesthetized, exsanguinated and fixed *in situ*. Exsanguination involved nicking the left ventricle and the right atrium. A cannula attached to a peristaltic pump was inserted into the left ventricle and the system flushed with ice cold PBS (1.5 ml/min, 10 min). Fixation was accomplished by perfusion with ice cold 4% formaldehyde/PBS (1.5 ml/min, 10 min). The aortic arch and carotid arteries were removed, fixed overnight in 4% formaldehyde/PBS, and embedded in paraffin.

### Morphometric Analysis

Whole tissue imaging of the arteries and implant was done using an Olympus SZ61 stereomicroscope equipped with an Olympus DP20 camera. Plaque length and device length were measured using digital calipers (Fisher Scientific).

### Histology & Immunohistochemistry

Plaques were sectioned beginning at the proximal end of the cast for 0.6–1.0 mm. Carotid cross-sections (7 µm, 4/slide) were mounted on SuperFrost Slides (Fisher Scientific) and sections at taken 0.1 mm intervals were stained with Masson's trichrome. Trichrome-stained slides were imaged on an Olympus BX51 microscope equipped with a Q Imaging Retiga 2000R digital camera and Slidebook software. The most stenotic section of the plaque (generally those sections most proximal to the margin) was used for comparison with UBM measurements. Percent stenosis was calculated with NIH ImageJ software using the following formula:
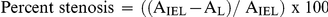
where A_L_ = the area of the lumen and A_IEL_ = the area circumscribed by the internal elastic lamina.

### Inter- and intra- observer variability of ultrasound measurements

Intra-observer variability was assessed by comparing the measurements of plaque length and percent stenosis from 8 different images made by the same investigator on 10 different days. Inter-observer variability was determined by two investigators independently analyzing the same set of images. Inter- and intra-observer variability was calculated as a percentage of the absolute difference between the two measurements divided by the mean of the two measurements [11].

### Statistics

Data and statistical analysis was preformed, and graphs were constructed, using Graph Pad Prism (V.5, San Diego CA) or Origin (v8.1, Northhampton, MA). For comparison of measures of the same variable by two techniques, the methodology of Bland and Altman [15] was used. Details of the analysis and results are presented with the data and figures. For time course experiments, data was analyzed by repeated measures analysis of variance (Statistica, v6, StatSoft Co, Tulsa, OK) with fixed within-animal effect of time, a fixed between-animal device group, and their interaction. The validity of t_S_/t_T_ and v_max_ as predictors of plaque formation was determined through generation of ROC curves discriminating between the presence (n = 27) or absence of plaque (n = 12).

## Results

### Choice of experimental conditions

In an effort to determine the applicability of UBM for detection of carotid atherosclerosis, we elected to test two constriction devices (cuff and cast), used both male and female mice, and assessed plaque development in both young (5 week) and older (17 week) mice.

### Implanted casts and cuffs are detectable by ultrasound

**Figure 2:**
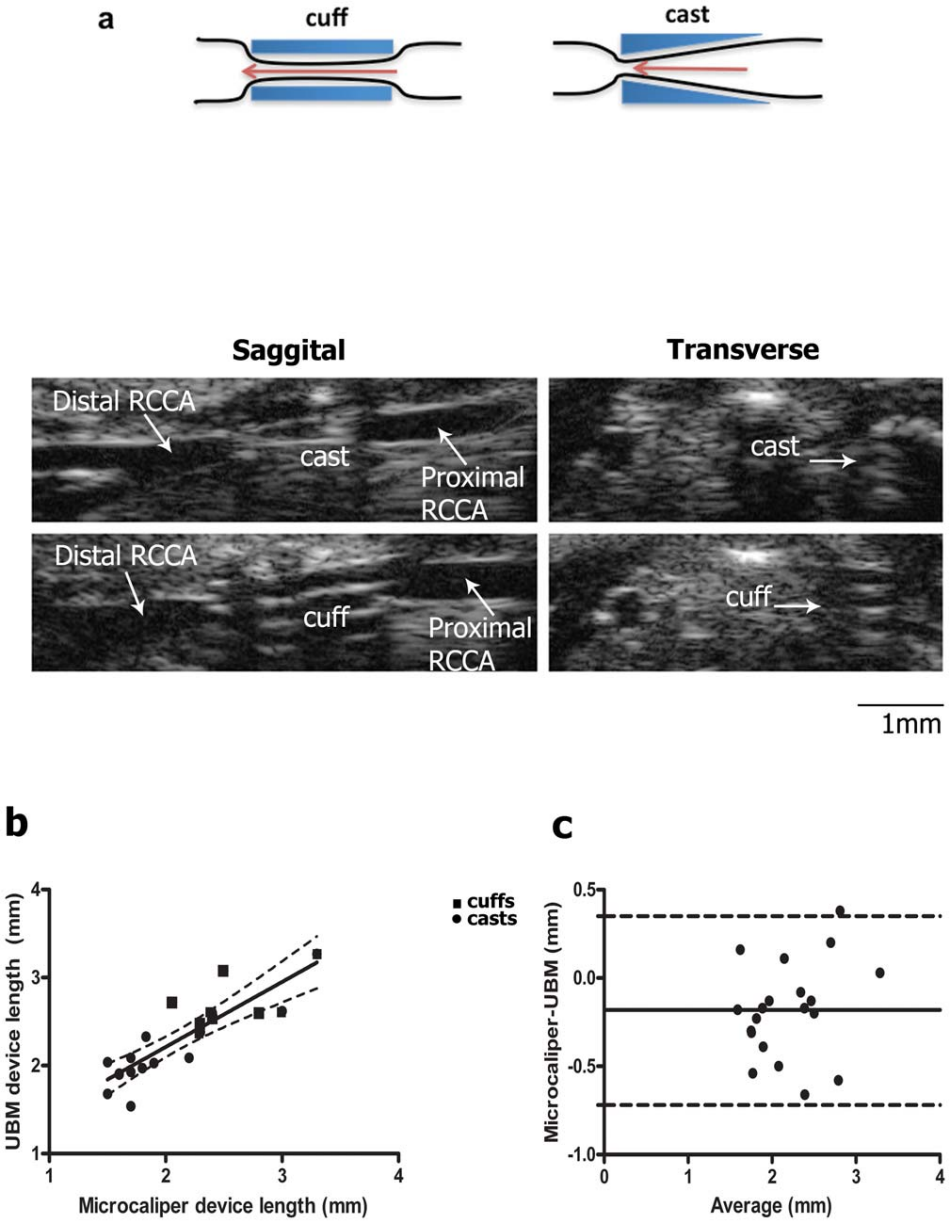
Implanted casts and cuffs produce a unique echogenic signature and their length can be precisely measured by ultrasound. Casts or cuffs were implanted around the right common carotid artery as described in Methods. (a) Representative sagittal and transverse B-mode ultrasound images of RCCA with cast or cuff 2 weeks post-surgery. Device length was measured from sagittal B-mode images or directly with microcalipers following fixation and the analyzed by (b) linear regression or (c) Bland-Altman Analysis. (b) Dotted lines indicate 95% confidence intervals. Correlation coefficient = 0.85; n = 20. (c) dotted lines: 95% limits of agreement. Bias, indicated by solid line, was calculated as -0.18 mm+/−0.27 SD.

Casts and cuffs produce unique echogenic signatures in both sagittal and transverse views (Fig. 2a). To determine the accuracy of UBM, the length of implanted cuffs and casts measured by UBM *in vivo* was compared with direct measurements of the excised devices following *in situ* fixation. The UBM measurements were not available to the individual analyzing the whole-mount preparations, making the measurements independent and blinded. The correlation between UBM and microcaliper measurements (r = 0.85, Fig. 2b) validated UBM as a reliable measurement tool for length in mouse carotid arteries. Bland-Altman analysis indicated that the differences between microcaliper and UBM measurements were relatively uniform over the length measured, with UBM measurements consistently longer (−0.184±0.27 mm, Fig. 2c). Intra- and inter- observer variability for ultrasound measurement was 3.6% and 1.5% respectively.

### Plaque progression can be quantified by ultrasound

**Figure 3:**
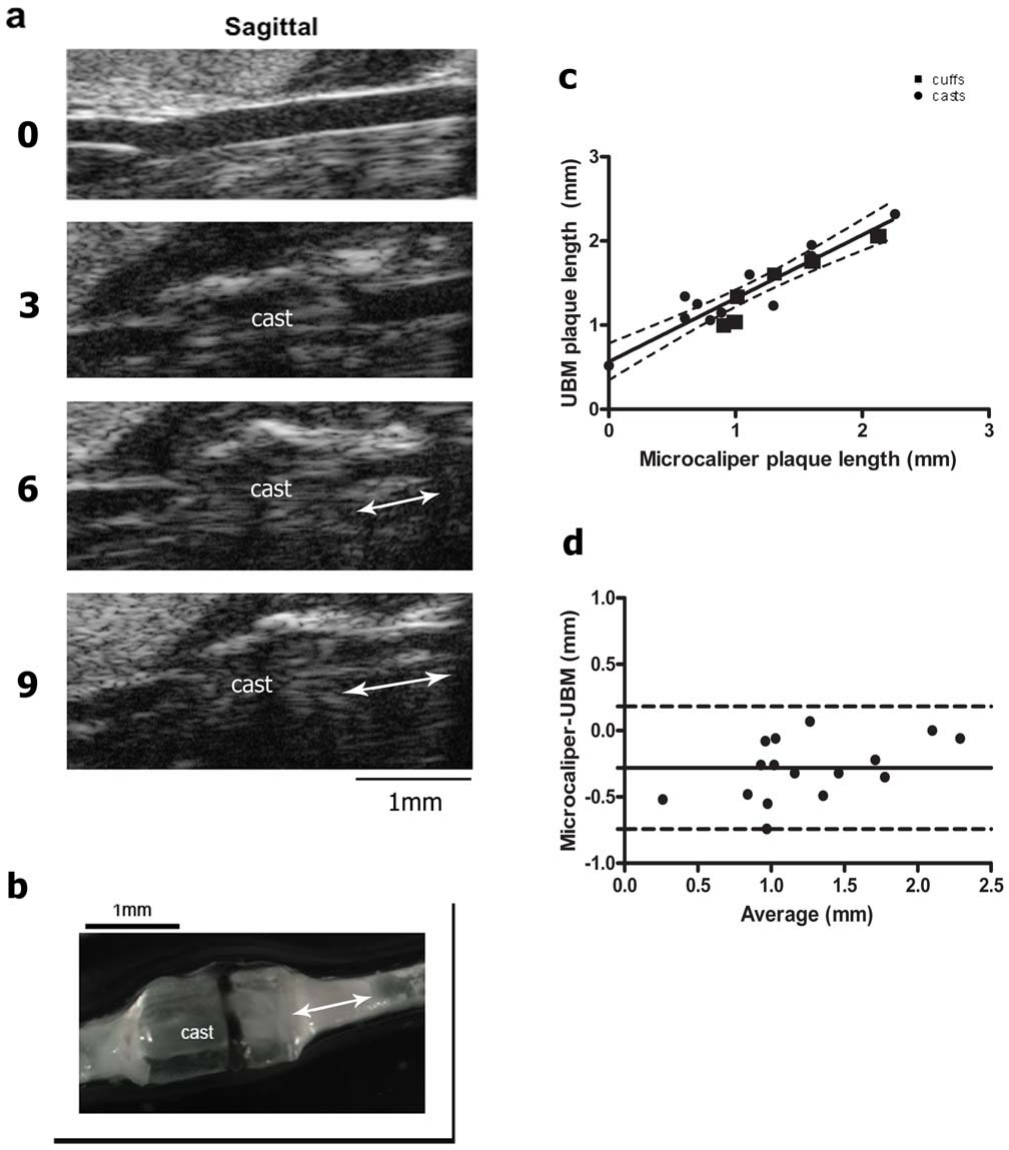
Plaque length is accurately measured by ultrasound. (a) Representative sagittal B-mode images of RCCA from one animal prior to surgery (0) and 3, 6 and 9 weeks after cast placement. Plaque length is indicated with arrows. (b) Whole mount of the carotid artery in (a) at study termination. (c) Linear regression of ultrasound vs microcaliper measurements of plaque length (n = 6 cuffs, 10 casts). Dotted lines indicate 95% confidence intervals. Correlation coefficient = 0.92, n = 16. (d) Bland Altman analysis of plaque length as measured by microcaliper and UBM. Dotted lines represent 95% limits of agreement. Bias, indicated by solid line, was calculated as −0.27 mm+/−0.22 SD.

Having established that UBM detected and accurately reported the length of the rigid devices, we tested the accuracy of UBM for detecting and measuring carotid plaques. B-mode saggital images of the right common carotid artery (RCCA) were taken prior to surgery. At that time, the artery walls were readily apparent as discrete bright white lines (Fig. 3a). The appearance of the artery wall was not noticeably different between pre-surgery and two weeks post-surgery, suggesting that neither the surgery nor the healing process significantly altered the artery architecture (data not shown). With time, the signal generated by the artery wall became more diffuse and the RCCA lumen increasingly compromised (Fig. 3a), features that, in humans, are diagnostic of plaques [16]. At 9 weeks post-surgery the carotid arteries were imaged by UBM. Plaques were defined as artery regions producing a diffuse UBM signal surrounding a compromised lumen (arrows, Fig. 3a) and their length determined using the UBM length measurement tools validated in Fig. 1. Following *in situ* fixation, micro-calipers were used to measure plaque length from whole-mount preparations (Fig. 3b, and as described above). The strong correlation (r = 0.92, n = 16; Fig. 3c) confirmed that the UBM signature does, in fact, reflect plaque. Thus, UBM accurately reported plaque in the live animals. Bland-Altman analysis indicated little bias and consistently small differences between UBM and microcaliper measurements; in addition, the small amount of bias was relatively consistent over a range of plaque lengths (Fig. 3d). UBM plaque measurements were consistently longer than those obtained from post-mortem whole-mounts (−0.29±0.23 mm). UBM intra- and inter-observer variability were 11.34% and 9.7%, respectively. In agreement with published studies [6], no plaque was apparent (either by UBM or in whole mounts) in the contra lateral artery, indicating that plaque formation was a result of carotid constriction (see Fig. S1).

### Stenosis can be determined by ultrasound

**Figure 4:**
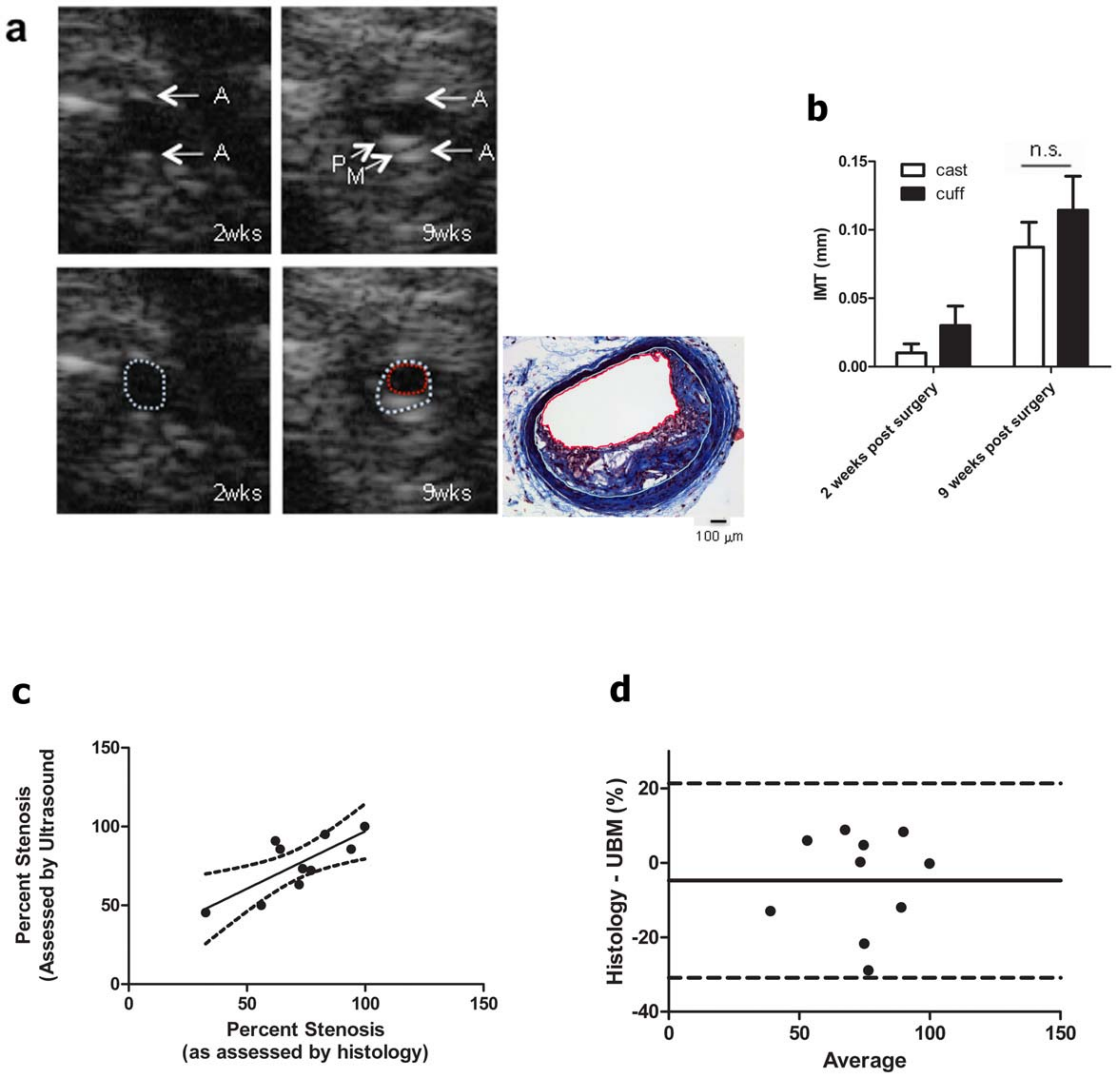
Percent stenosis calculated from UBM parameters correlates with histological measurements. (a) Transverse B-mode image of RCCA 2 and 9 weeks after surgery. Upper Images, Adventitia is marked “A”, plaque “P”, “Medial layer M”. Lower, images are identical, but the inner elastic lamina is outlined with dotted white line, lumen with a red dotted line. Trichrome-stained transverse sections of the same 9 wk artery; internal elastic lamina (IEL) and lumen are outlined in white and red, respectively. (b) IMT measurements of casted and cuffed animals after 2 and 9 weeks of surgery. n.s. Not significant. (c) Linear regression analysis of percent stenosis estimated by UBM and histology. See Materials and Methods for calculations of percent stenosis. Dotted lines indicate 95% confidence intervals; correlation coefficient = 0.75, n = 10 casts. (d) Comparison of UBM and histology measurements using Bland-Altman analysis. Dotted lines indicate 95% limits of agreement. Bias is indicated by solid line, was calculated as −4.76 mm±13.3 SD.

The accuracy of UBM for determination of percent stenosis was investigated using transverse images of the carotid. Prior to surgery, the adventitia was visible as two bright white lines (Fig. 4a, labeled “A”) while the intimal and medial layers were not discernable. With time, the lumen becomes compromised by the plaque (Fig. 4a, “P”). The thin non-echogenic space between the plaque and the adventitia represents the medial layer (Fig. 4a, “M”). Using these landmarks, the internal elastic lamina (IEL) and lumen can be outlined (Fig. 4a dotted lines). To evaluate changes in arterial remodeling, the area of the lumen, IEL and outer artery were measured over time (Table 1). Our results show that 9 weeks post-surgery, the artery lumen diameter had decreased, while the area circumscribed by the IEL had significantly increased, evidence of both inward and outward remodeling. To evaluate plaque burden, intima to medial thickeness (IMT) was measured. Both cast and cuff constriction increased IMT with time (Fig. 4b). Percent stenosis was calculated from 9 week transverse images using the IEL and lumen areas (as detailed in Materials and Methods). Percent stenosis determined from UBM was compared to that calculated from trichrome-stained sections of the same region using the device margin as a reference point. The correlation (r = 0.75) between UBM and histology (Fig. 4c) validated the accuracy of UBM for determination of carotid stenosis in live animals. Bland-Altman analysis revealed that the little bias there was was relatively consistent over a broad range (Fig. 4d).

**Table 1:** Changes in Lumen and IEL area upon carotid constriction.

	Lumen Area(mm^2^)	IEL Area(mm^2^)	Neointima Area (mm^2^)IEL Area – Lumen Area
CAST (n = 10)			
Pre-Surgery	0.08±.01	0.08±.01	0.00±0.00
2 weeks post surgery	0.09±0.01	0.10±0.01	0.01±0.01
9 weeks post surgery	0.04±0.01* ^,^ †	0.16±0.01* ^,^ †	0.12±0.01* ^,^ †
CUFF (n = 7)			
Presurgery	0.10±.01	0.10±.01	0.00±0.00
2 weeks post surgery	0.11±0.01	0.12±0.01	0.01±0.01
9 weeks post surgery	0.08±0.02	0.14±0.01*	0.06±0.01* ^,^ †

Data was analyzed by One Way ANOVA.

“*”indicates p<0.05 as compared to presurgery.

“†” indicates p<0.05 as compared to 2 weeks post surgery.

### Determination of Rate of Plaque Progression

**Figure 5:**
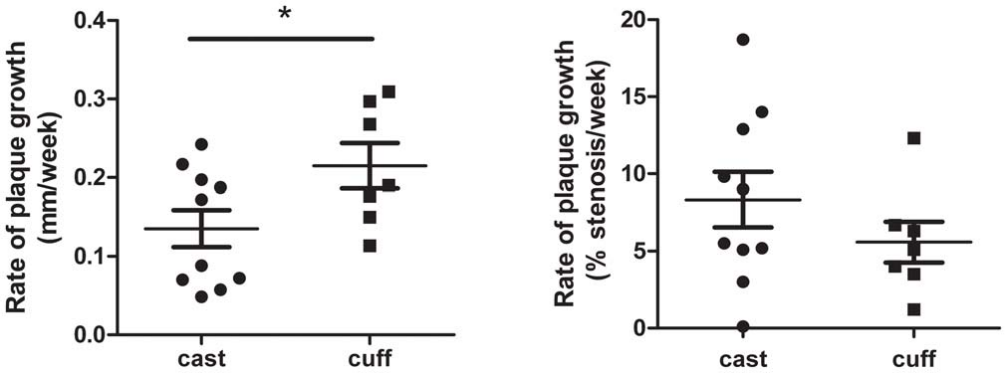
Plaques Progress More Rapidly in Casted vs Cuffed Animals. The rate of plaque growth was determined by performing individual linear regression analysis of plaque length or stenosis over time for each animal. The slopes (rates) were compared by unpaired t-test. (a) Rate of plaque growth in length (b) Rate of plaque growth in terms of stenosis. Data are mean±SEM, n = 7 cuffs, 10 casts “*” indicates p<0.05. (See Fig. S2 for source data.)

As UBM is non-invasive, plaque length and stenosis can be followed in individual animals using repeated measures, with the slope of the resulting graph a measure of the rate of plaque development (length and stenosis, respectively). Despite their genetic identity, the rate of plaque growth and development of stenosis in individual mice was variable (Fig. S2). To determine if the type of constriction influenced plaque development, linear regression analysis of plaque length or stenosis over time was done for each animal. The slopes (rates) were then compared by unpaired t-test. This analysis revealed a significantly higher rate of plaque growth in length in cuffed vs. casted arteries (Fig. 5a); the development of stenosis was not significantly different (Fig. 5b).

### Velocity Parameters Predict Plaque Development

**Figure 6:**
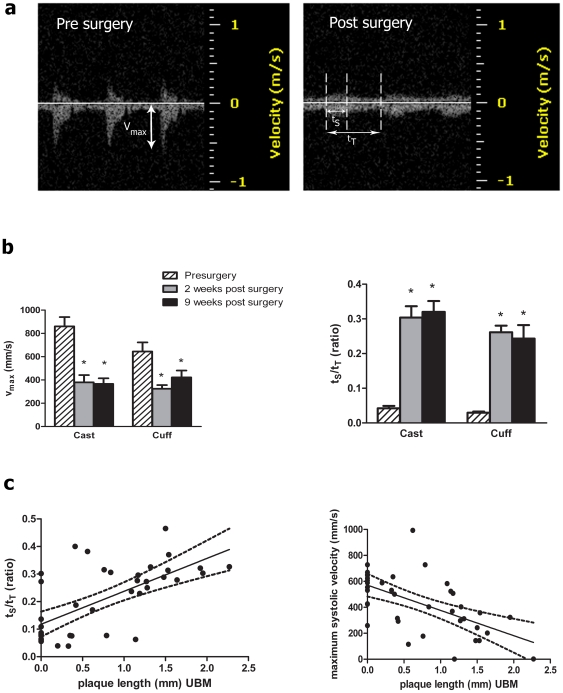
Pre-plaque PW-Doppler parameters correlate with plaque length at 9 weeks. (a) Representative PW-Doppler tracing of a proximal RCCA before and after surgery. Note the dramatic decrease in velocity. Measurements of v_max_, time in systole (t_s_) and t_T_ (total time) are indicated. (b) v_max_ and t_s_/t_T_ were calculated from pre-surgery tracings and 2 and 9 weeks after device placement. Data (mean±SEM, n = 7 cuffs, 10 casts), were analyzed by 2 way ANOVA. There was a highly significant effect of device on v_max_ and t_s_/t_T_ ratio (p<0.005), but no significant difference between cast and cuffs on either parameter. “*” p<0.05 compared to pre-surgery. (c) Using 39 animals fitted with casts, 2 week t_s_/t_T_ and v_max_ values were calculated and plotted against plaque length at 9 weeks and the correlation determined by linear regression. t_s_/t_T_ positively correlated with plaque length (p<0.0005, r = 0.66) between, v_max_ and plaque length were negatively correlated (p<0.0005, r = 0.59). Dotted lines indicate 95% confidence interval.

While changes in blood flow predispose areas of the vasculature to atherosclerosis, a correlation between specific initiating blood velocity parameters and resulting plaque size has not been investigated. Indeed, until the carotid constriction model, it has not been possible to reproducibly modulate carotid blood flow in mice *in vivo*. Blood velocity in the carotid artery was examined using PW Doppler. A comparison of the PW Doppler velocity tracings pre-, and two weeks post-, surgery revealed that both devices significantly reduced the maximum systolic blood velocity (v_max_) in the artery proximal to the device (Fig. 6a and b). This reduction was accompanied by a broadening of the systolic velocity peak (Fig. 6a and b), characteristic of disturbed flow [17]. Peak broadening was quantified as an increase in the duration of the systolic velocity peak (t_S_) over total time between peaks (t_T_). Alterations in these parameters did not differ significantly between casts and cuffs, indicating similar blood flow modulation by both devices. Noting that carotid constriction altered proximal blood velocity, we asked how well the PW-Doppler v_max_ and t_S_/t_T_ measurements, taken two weeks post-surgery, correlated with plaque length at 9 weeks post-surgery. These studies were done using cast constriction and the sample size was increased to 39 animals. Linear regression analysis revealed a positive correlation between t_S_/t_T_ ratio and overall plaque length at study termination (p<0.0001, r = 0.658) (Fig. 6c). Similarly, we found a negative correlation with v_max_ and plaque length at two weeks (p<0.0001, r = 0.588) (Fig. 6c). Of particular interest was determination of the values of v_max_ and t_S_/t_T_ that best predicted plaque development. ROC curves were generated to determine the cutoff value of the t_S_/t_T_ ratio or v_max_ that would best predict the velocity profile below which plaque would not form. ROC curves were generated using the pathological determination of plaque as the outcome and the ultrasound parameter at two weeks as the test measurement potentially predicting plaque. The cutoff value for the ultrasound parameter was then varied in order to determine the value at which the sensitivity and the specificity are optimal. The results revealed that both t_S_/t_T_ ratio and v_max_ were fairly accurate predictors of plaque formation (Table 2). Further, the ROC curves suggested that the best prediction using t_S_/t_T_ could be obtained by using a cutoff value for the t_S_/t_T_ ratio of 0.15 with values >0.15 predicting plaque formation with a specificity and sensitivity of 83.3% and 80% respectively. Similarly, v_max_<530 mm/s predicted that a plaque would form with a specificity and sensitivity of 75% and 77.8% respectively. Post mortem analysis of animals with t_S_/t_T_ ratio <0.15 or v_max_>530 revealed that the sutures around the device had loosened after surgery, accounting for the insufficiently disturbed blood flow and absence of plaque. As suture loosening was not apparent from the B-mode images, the blood flow parameters provided an additional level of quality control.

**Table 2:** Sensitivity and Specificity of PW-Doppler parameters t_s_/t_T_ and v_max_ as predictors of plaque formation.

	t_s_/t_T_	v_max_
Sensitivity (%)	83.3	75
Specificity (%)	80	77.8
Area under curve	0.8	0.8
95% Confidence Interval	0.67 to 0.94	0.65 to 0.95
Cut-off value	>0.15	<530
p-value	<0.005	<0.005

The results from this study validate the use of UBM to predict, measure, and follow carotid plaque development (ie, length and percent stenosis) in mice with time. As a non-invasive tool, UBM affords the power of repeated measures, permitting rate determinations to be calculated for individual animals and enabling investigators to study the effects of genetic or pharmacological manipulations on the rate of plaque development. Additionally, a set of PW criteria (v_max_ and t_S_/t_T_) have been defined that predict plaque formation.

## Discussion

Although mouse models of atherosclerosis have greatly contributed to our understanding of the disease process, most have studied plaques in the descending aorta, aortic root, and aortic arch, locations prone to plaque formation in mice but of limited applicability to the carotid atherosclerosis in humans. Two groups have reported that carotid plaques can be induced in Apo E−/− and LDLR−/− mice by carotid constriction[3], [4], [6]. On the Apo E−/− background, the plaques share many features with vulnerable carotid plaques in humans including morphology, accumulation of lipids, regions of necrosis, the presence of macrophages, and intraplaque hemorrhage [4]. Thus, Apo E−/− was the strain of choice for our studies. For constriction, one group uses a cuff cut from silastic tubing [3] and the other employs a molded conical cast [4]. As the cuffs are hand-cut and the casts are commercially molded, the cast size and dimensions are more consistent (Fig. 2b). Given that both devices similarly constrict blood flow and induce plaques, the casts provide a more reproducible constrictive device.

As atherosclerosis is an inflammatory disease [18], it was important to establish that plaque induction was dependent on reduced blood flow and not an artifact of surgical manipulation. Several lines of evidence support the dependence of plaque formation on decreased blood flow: 1) no carotid plaques were induced in animals exposed to sham surgeries, in which the carotid artery was exposed and manipulated but no device was implanted and 2) implantation of a larger diameter non-constrictive device (either cuff or cast) did not alter blood flow or induce plaques (data not shown). Particularly informative were the study animals that had flow two weeks post-surgery but never formed plaques (Fig. 6 c and d). A detailed examination of the UBM in these non-plaque animals revealed that post-surgery blood flow was similar to pre-surgery (high v_max_ and low t_S_/t_T_), due to misplacement of the device, loosening of the sutures, or a device that had come off. Thus, with all other criteria being comparable (ie, surgery, suturing, presence of the device, inflammation due to device implantation, etc) plaques formed only when the blood flow was disturbed, providing convincing evidence that it is the blood flow, rather than the surgery, foreign body response, or healing process, that induced carotid plaques.

### UBM provides quality control

The fact that cuffs and casts generate unique UBM signatures (Fig. 2) allowed us to visualize device placement and provided a landmark for consistent imaging. The resolution of the UBM images, as well as the reproducible changes in blood flow, enabled us to detect complications early in the study. Post-surgery UBM was started two weeks following surgery, allowing time for the incision to completely heal. At this time, there were no detectable changes in the appearance of the artery (compared with pre-surgery). At two weeks post-surgery, blood flow both proximal and distal to the device was detected in the majority (cuffs: 84.6%, casts: 77.7%) of animals. Generally speaking, animals with blood flow two weeks post-surgery maintained flow throughout the subsequent 7 weeks in the study (cuffs: 8/11, casts 13/14). B-mode imaging detected several types of complications, including a cuff that had come off the artery after surgery and, most often, vessel occlusion that blocked blood flow. Post-mortem examination confirmed artery occlusion resulting from either a blood clot or improperly positioned device. Thus, B-mode and PW-Doppler data two-weeks post-surgery enables one to detect complications (ie, vessel occlusion or no constriction) that required exclusion of the animals from the study and ensuring that all animals completing the study have similar blood flow patterns.

### UBM determinations parallel histological measurements

Our results demonstrate that, compared with direct whole mount measurements, UBM accurately reports plaque length over a range of 0.6–2.2 mm (Fig. 3). Of note, ultrasound length measurements were consistently longer than those obtained post-mortem (bias = −0.27±0.23 mm). Gan et al. noted a similar discrepancy in the ascending aorta, suggesting that fixation may cause tissue contraction [10]. However, because we see some bias in the measurements of the devices (−0.18±0.27), which should not contract, there is likely a contributing bias in one of the measuring tools (UBM or microcalipers).

Plaques were visualized in saggital and transverse planes. Transverse views provide a more accurate image as, in sagittal views, stenosis can be misinterpreted due to plaque eccentricity, an off-axis scan, or acoustic shadowing [17]. Prior to surgery, only the advential layer was visible by UBM. This may be due to limitations in the resolution of the ultrasound, or the fact that normal mouse intima is thin, consisting of an endothelial monolayer. In contrast, human intima contains smooth muscle and connective tissue fibers [19]. It is possible that the 55 MhZ probe may have sufficient resolution to detect the mouse intima. By 9 weeks, plaque was apparent within the lumen. Intimal and medial layers could be identified, and IMT measurements showed an increase in thickness with time. To assess the contribution of inward and outward remodeling, both luminal are and area within the IEL were measured. Over time, luminal area decreased, while the area within the IEL increased, indicating components of both inward and outward remodeling of the vessel. This finding agrees with studies of human atherosclerosis demonstrating that outward remodeling of the vessel can occur as an initial compensatory mechanism of remodeling [20].

Percent stenosis was also calculated using IEL and lumen area measurements obtained by UBM. UBM calculations of percent stenosis correlated with those obtained histologically (range: 35%–100% stenosis) (Fig. 4). Bland-Altman analysis of these measurements revealed relatively little bias between UBM and histological measurements.

### UBM as a non-invasive tool to study plaque progression

Atherosclerosis develops slowly, with advanced lesion development requiring 12–20 weeks of high fat diet, or longer (7–11 months) with normal chow [19]. Traditionally, animals are sacrificed at a defined time-point and their plaques assessed histologically. However, not all animals will be at the same stage of plaque development at a given time (Fig. S2), necessitating the use of large numbers of animals to obtain sufficient material at a defined stage. Herein we establish the limits of sensitivity of UBM for detection of length, by comparing measurements of the implanted devices (Fig. 2) as well as plaques (Fig. 3) with those obtained using microcalipers. Additionally, we exploited the non-invasive nature of UBM to determine rates of plaque development, as defined by changes in length and stenosis with time. The determination of rate by individual linear regression analysis for each animal takes into account the biological variability between animals. Interestingly, animals fitted with cuffs developed plaques faster than those fitted with casts, despite similar flow generation. This may be due to gender differences, females reportedly developing plaques faster than males, although this is somewhat controversial [21]. While the effect of the rate differences on plaque composition or stability was not investigated in these studies, the ability to measure subtle changes in plaque size and stenosis becomes important in such contexts as assessing the effect of experimental drugs or genetic manipulations on plaque progression.

### Plaque Formation can be Predicted from Pre-Plaque UBM Parameters

PW-Doppler detected dramatic changes in post-surgery blood flow. As noted by Ding and colleagues (and confirmed in our studies, Figs. 6a and b), cuff placement results in a decrease in v_max_ in the area proximal to the device. Upon closer examination, we noted an extension of the time spent at this velocity, quantified as in increased t_S_/t_T_ ratio. As determined by our ROC analysis, animals exhibiting a low t_S_/t_T_ ratio (<0.15) or elevated v_max_ (>530 mm/s) rarely developed plaques. Thus, these cut-off values for v_max_ and t_S_/t_T_ ratio provide insight into the level of flow disturbances required for plaque formation. Additionally, post mortem analysis of animals with these flow patterns confirmed the absence of plaque and revealed that the sutures around the device had loosened after surgery, accounting for the lack of disturbed blood flow and absence of plaque. As suture loosening was not apparent from the B-mode images, the blood flow parameters provided an additional level of quality control.

### Study Limitations

To determine the extent to which UBM can be used to assess carotid plaque development, we tested male and female, young and old, mice and tow devices for artery constriction (cuffs and casts). Regardless of the parameters, UBM measurements correlated well with the histology, the current gold standard for plaque assessment. Thus, we have shown that a variety of plaque parameters (length, stenosis, IMT) can be determined non-invasively, making repeated measures on the same animal possible. *It should be noted that these studies were done with a 40 MHz transducer; a 55 MHz probe is now available and should improve the resolution of the measurements*. In contrast to analysis by histology, UBM requires fewer animals and rate determinations can be made. While it is true that artery constriction accelerates plaque progression in mice, this model is not unlike other mouse models of chronic human diseases, most notably collagen induced arthritis and experimental autoimmune encephalomyelitis (EAE). In these models, susceptible mice (DBA/1 (H-2^q^) and SJL, respectively) are injected with proteins (collagen, myelin derived peptides) that induce human-like disease over a period of weeks-months. Similarly, our model takes susceptible mice and uses carotid constriction to accelerate plaque development. Although there are differences in the hemodynamics of human vs mouse circulation, carotid constriction in mice produces UBM blood flow patterns very similar to those in humans and plaques with similar composition (foam cells, cholesterol crystals, thin fibrous cap, necrotic regions [3], [4]. Thus, the mouse model recapitulates flow-induced carotid atheroslcerosis, although with an accelerated timeframe. Mouse models are valuable as long as one is cognizant of their limitations.

In summary, we have demonstrated the ability of UBM to 1) detect cuffs and casts placed around the carotid artery, 2) confirm the correct placement and constriction of the devices, 3) accurately report plaque length and stenosis, 4) calculate the rate of plaque development, and 5) predict, at two weeks post-constriction, the probability of future plaque formation. The use of different constrictive devices, different ages, and different sexes of mice confirm that UBM is widely applicable to the study of carotid atherosclerosis. Finally, we have validated UBM as a non-invasive method for assessing plaque status in live animals. Such a tool is invaluable for determination of the effects of different genetic backgrounds, drug treatments, etc on plaque progression, measurements that, until recently have not been possible in a carotid plaque model.

## Supporting Information

Figure S1 **Absence of plaque in contra-lateral artery of animals with constrictive devices.** Representative whole mounts of aortic arch and common carotid arteries 9 weeks post-surgery from animals implanted with a cuff or cast. Despite plaque in the arch and subclavian artiery, the contra-lateral artery had no evidence of atherosclerosis. Representative of 7 cuffed and 39 casted animals.
(TIF)

Figure S2 **Biological variability of carotid plaque progression in animals fitted with casts or cuffs.** Plaque length and percent stenosis were measured over time in ApoE−/− animals fitted with cast or cuff. Measurements from individual animals are shown, n = 7 cuffs, n = 10 casts.
(TIF)
