# Supplementary figures and images for: Ultrasound Biomicroscopy for Longitudinal Studies of Carotid Plaque Development in Mice: Validation with Histological Endpoints
Source: PLoS One. 2012 Jan 5;7(1):e29944. doi: 10.1371/journal.pone.0029944 (PMC3252361; doi:10.1371/journal.pone.0029944)

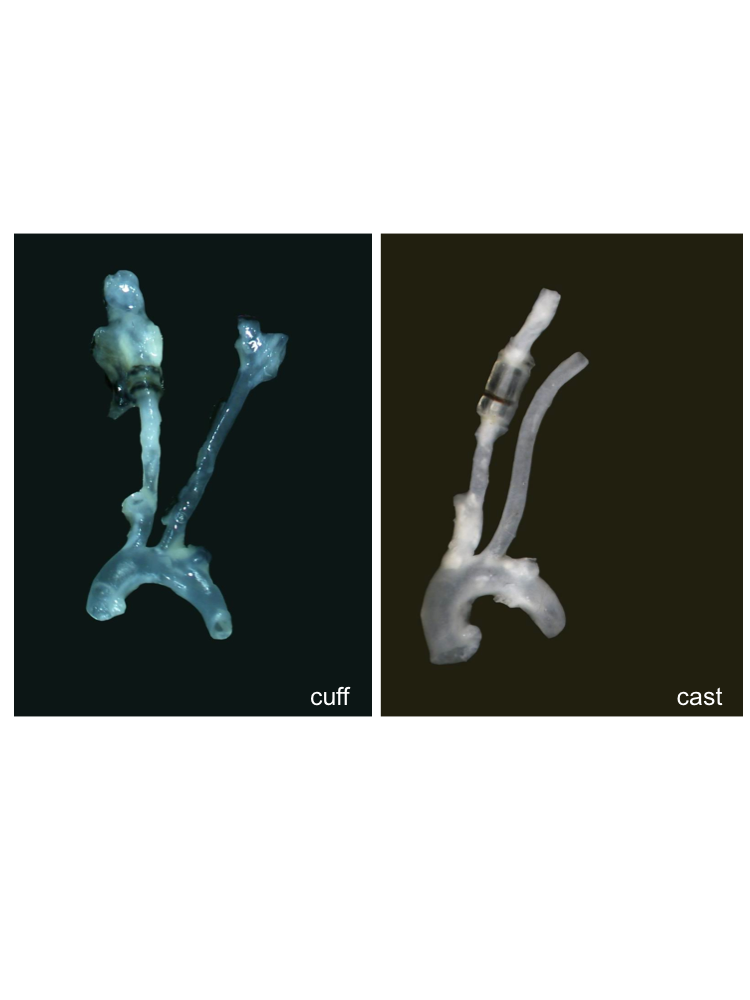

Supplement: Figure S1 — Absence of plaque in contra-lateral artery of animals with constrictive devices. Representative whole mounts of aortic arch and common carotid arteries 9 weeks post-surgery from animals implanted with a cuff or cast. Despite plaque in the arch and subclavian artiery, the contra-lateral artery had no evidence of atherosclerosis. Representative of 7 cuffed and 39 casted animals. (TIF) [file pone.0029944.s001.tif]

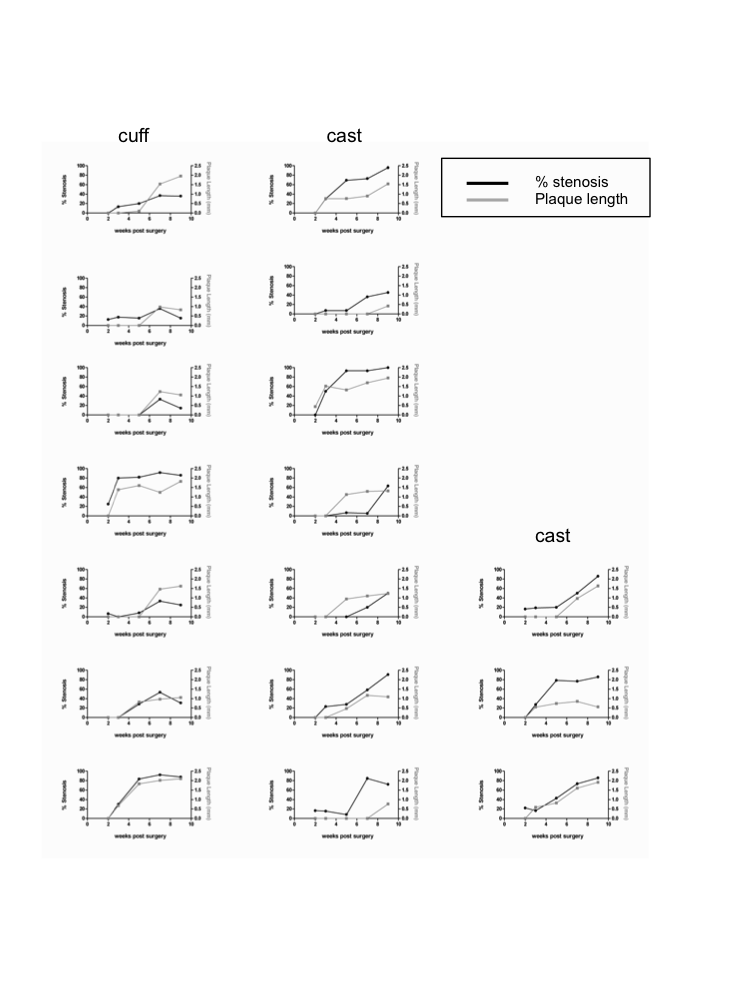

Supplement: Figure S2 — Biological variability of carotid plaque progression in animals fitted with casts or cuffs. Plaque length and percent stenosis were measured over time in ApoE−/− animals fitted with cast or cuff. Measurements from individual animals are shown, n = 7 cuffs, n = 10 casts. (TIF) [file pone.0029944.s002.tif]
